# Whole transcriptome sequencing reveals a TCF4-ZNF384 fusion in acute lymphoblastic leukemia

**DOI:** 10.3389/fonc.2022.900054

**Published:** 2022-08-16

**Authors:** Zhengyu Wu, Fang Zhang, Chengzhu Liu, Shuhong Shen, Jinhua Chu, Linhai Yang, Zhiwei Xie, Yu Liu, Kangkang Liu, Ningling Wang

**Affiliations:** ^1^ Pediatrics, the Second Affiliated Hospital of Anhui Medical University, Hefei, China; ^2^ Pediatric Translational Medicine Institute, Shanghai Children’s Medical Center, Shanghai Jiao Tong University School of Medicine, Shanghai, China; ^3^ Key Laboratory of Pediatric Hematology & Oncology Ministry of Health, Department of Hematology & Oncology, Shanghai Children’s Medical Center, Shanghai Jiao Tong University School of Medicine, Shanghai, China

**Keywords:** acute lymphoblastic leukemia, ZNF384, RNA sequencing, TCF4, pediatic

## Abstract

Previous studies have shown that, the clinical features and prognosis of ZNF384-rearranged pediatric acute lymphoblastic leukemia (ALL) depend on its translocation partners. We report two cases of TCF4-ZNF384 fusion, one 6-year-old girl and one 10-year-old boy, both diagnosed by whole-transcriptome sequencing, and TCF4 is the newest fusion partner of ZNF384. As illustrated in this first report of TCF4-ZNF384 fusion in ALL patients, the identification of patients with ZNF384 rearrangement in ALL patients is critical to elucidate outcomes associated with a specific rearrangement and to develop appropriate treatment strategies. In addition, the development of other methods to detect ZNF384 specific translocation partners and leukemia specific targeting agents is of great significance to further improve the prognosis of ALL with ZNF384-rearrangement.

## Introduction

Acute lymphoblastic leukemia (ALL) is the result of clonal proliferation of lymphoid stem cells or progenitor cells, of which more than 80% are derived from B cells (B-ALL) (1). Cytogenetic and molecular biological abnormalities play a key role in the pathogenesis of ALL by affecting cell differentiation, cell cycle, tumor inhibition, and stem cell self-renewal (2). The detection of these abnormalities can facilitate diagnosis, risk stratification, and targeted therapy. Next generation sequencing (NGS) has identified several novel subtypes of ALL, including the ZNF384 rearrange-but interestingly, patients with this subtype appear to express a variety of leukemia phenotypes, including B-ALL (myeloid markers with or without abnormal expression) and B/myeloid mixed phenotype leukemia (3). In this regard, ZNF384 rearrangement can be detected in 3-5% of ALL children, and the clinical features of these children is often determined by the fusion partner of ZNF384 rather than by ZNF384 itself (3–5). More than 9 fusion partners of ZNF384, such as EP300, EWSR1, TCF3, CREBBP and TAF15, have been identified by RNA sequencing and conventional methods, but the significance of each fusion partner has not been clarified due to the small number of reported cases (5).

Recently, TCF4 has been identified as a new fusion partner of ZNF384. Here we describe two children with *TCF4-ZNF384* rearrangement, a novel fusion that has not been reported to date (6, 7).

## Materials and methods

### Case presentations

Case-1 was a 10-year-old boy who was admitted to hospital with bone pain and sallow complexion for more than 1 month. Blood cell count indicated: white blood cell 18.4×10^9^/L, hemoglobin 50g/L, platelets 111×10^9^/L. Bone marrow puncture showed that a group of primitive cells accounted for about 82.5% of the nuclear cells. Histochemical PAS staining (+), histochemical POX (-). Flow cytometry analysis of bone marrow samples showed that CD19 (91%), cCD79a (43%), CD10 (2%), CD20 (1.4%), CD22 (77%), CD33 (6%), CD116 (21%), CD71 (46%), CD34 (98%), HLA-DR (84%), CD117 (12%), CD38 (97%) were positive, cCD22, CD21, TdT, MPO, CD13were negative. Case 1 received chemotherapy according to CCCG-ALL-2020 protocol (Chinese Cancer Cooperative Group) and achieved complete hematological response (CR) after induction therapy. MRD of bone marrow was detected on Day19 and Day46 (MRD<0.01%), the total course of treatment is not yet finished, and the CR status has been maintained for more than 11 months. There were no serious complications during the treatment (**Table 1**).

**Table 1:** Clinical characteristics of ALL with TCF4-ZNF384 fusion genes.

ID	Sex/Age (years)	Initial WB Ccount	Karyo type	Initial risk	CNS involvement	Current status	Status	FUP (years)
Case-1	M/10y	18.40×10^9^/L	46, XY	IR	CNS3	1^st^CR	Alive	1.5
Case-2	F/6y	10.73×10^9^/L	46, XX	SR→IR	CNS1	1^st^CR	Alive	5

F: female; M: male; WBC: white blood cell; CNS: central nervous system; FUP: follow up; SR, standard-risk; IR: intermediate-risk; CR: complete remission.

Case-2 was a 6-year-old girl. Blood cell count indicated: white blood cell 10.73×10^9^/L, haemoglobin 101g/L and platelets 54×10^9^/L. Bone marrow puncture showed that a group of primitive cells accounted for about 90.8% of nuclear cells, histochemical PAS staining (+), histochemical POX (-). Flow cytometry analysis of bone marrow samples showed that CD19 (69.5%), cCD79a (66.9%), cCD22 (86.9%), CD22 (99.5%), TdT (46%), CD33 (90%), CD34 (99.5%), HLA-DR (83.8%), CD9 (28.8%), CD123 (52.4%), CD38 (89.1%) was positive and CD10, CD20, CD21, CD13 and MPO were negative. Case 2 received chemotherapy according to CCCG-ALL-2015 protocol and achieved complete hematological response (CR) after induction therapy. MRD of bone marrow was monitored 0.17% on Day19 and 0.02% on Day46. The first course of VDLP (Vincristine + Prednisone +Daunorubicin + L-asparaginase) induced poor remission but MRD of follow-up treatment was < 0.01% (once every 6 months). At present, the drug has been stopped for more than 2 years, and the CR status has been maintained for more than 24 months (**Table 1**).

### Karyotype analysis

White blood cells extracted from patient bone marrow specimens were cultured (24 and 48 h, without stimulation), collected, and G-zone slides were prepared using standard cytogenetic techniques according to specimen specific protocols.

### RNA sequencing

Ribozero’s method was used to remove the ribosomal RNA from the total RNA and then reverse transcription into cDNA, which was used as a template to construct a library supporting sequencing. Illumina Hiseq X sequencing platform was used to test the whole transcriptome level of THE RNA samples of the subjects. Bioinformatics analysis of gene mutations and gene fusion: The sequenced fragments were compared with the UCSC HG19 reference genome by STAR software. Mutation detection was performed using MuTect2 software. Fusion Catcher was used for gene fusion prediction.

## Results

**Figure 1:**
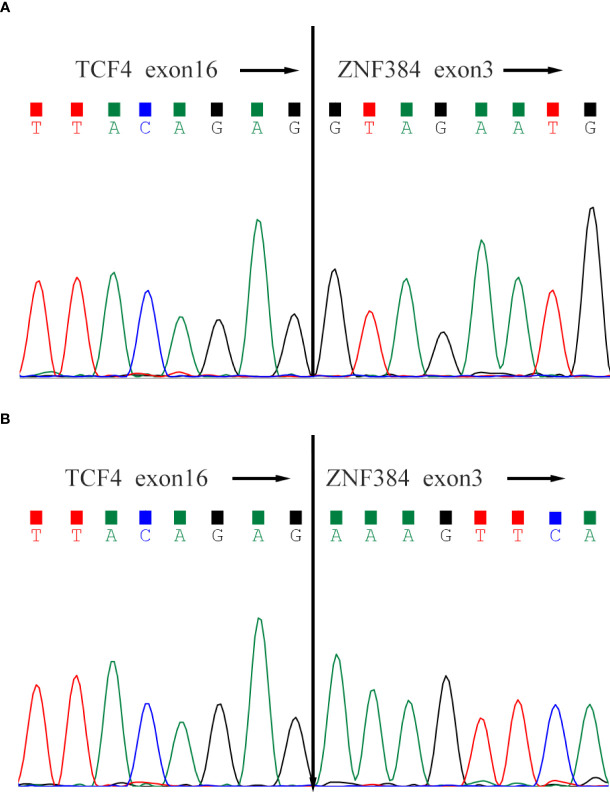
Sanger sequencing of the TCF4-ZNF384 fusion transcript. Both patients' breakpoints were located at exon 16 of TCF4 and exon 3 of ZNF384, **(A)** for Case-1 and **(B)** for Case-2.

**Figure 2:**
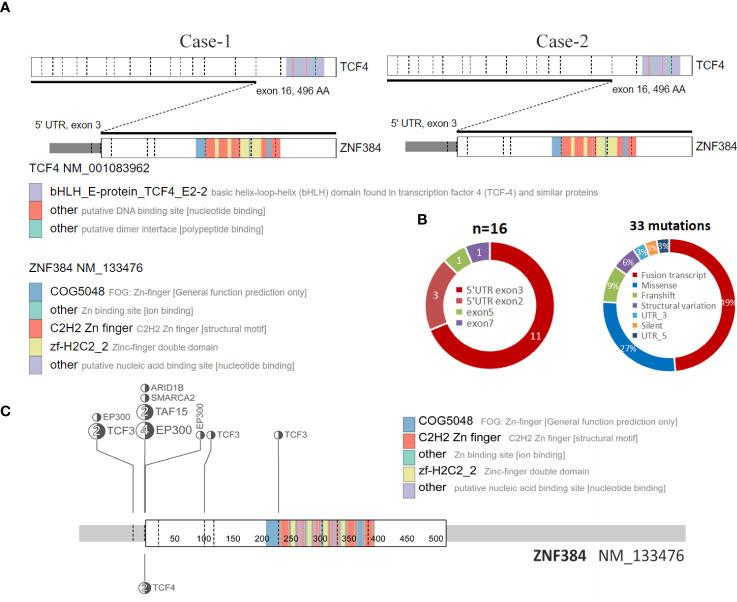
Structure of the ZNF384-related fusions. **(A)** The fusion of TCF4::ZNF384 and TCF4::ZNF384 chimeric protein was produced in case 1-2. Conserved protein domains (predicted by Pfam, https://pfa-m.xfam.org/) are color coded and shown in the accompanying key. Protein diagrams were generated using ProteinPaint (https://proteinpaint.stjude.org/). **(B)** In 33 samples with ZNF384 mutations, nearly half were ZNF384 fusion transcripts. Among these fusion partners, EP300 and TCF3 were the most common, while the fusion breakpoints of ZNF384 were mainly concentrated in the 5' UTR exon3 region, which almost retained the complete functional domain of ZNF384[5].(https://proteinpaint.stju-de.org/). **(C)** Summary of all TCF4::ZNF384 fusion cases described to date.

In case-1, conventional karyotyping showed that all 20 cells were normal male karyotype (46, XY), RNA.seq detected the presence of *TCF4-ZNF384* fusion, and the 5’-end gene breakpoint was located at Chr18:52901775, and the 3’-end gene breakpoint was located at Chr12:6788691. In addition, ASXL1 W583fs mutation (mutation frequency: 37.9%) and FLT3 A680V mutation (mutation frequency: 10.7%) were also detected. In case 2, conventional karyotyping showed that all 20 cells were normal female karyotype (46, XX), RNA.seq detected the presence of *TCF4- ZNF384* fusion, and the 5’-end gene breakpoint was located at Chr18:52901779, and the 3’-end gene breakpoint was located at Chr12:6788691. In addition, FBXW7 mutation was also detected (**Figures 1**, **2**).

## Discussion

To the best of our knowledge, this is the first report of two patients with ALL with a TCF4-ZNF384 fusion. In recent Some scholars have suggested that it could be used as a separate group of B-ALL genetic credit types. In the fusions of ZNF384 discovered to date, the fusion breakpoint of ZNF384 tends to be concentrated in the 5 ‘UTR region, while the protein domain of ZNF384 is intact (8), which is also present in these two patients. Among all of the cases, most functional domains of TCF4 were lacking in the resulting fusion proteins, while the complete ZNF384 protein was retained (**Figure 2A, C**). However, the role of this fusion in ALL is still unclear due to its rarity.

**Figure 3:**
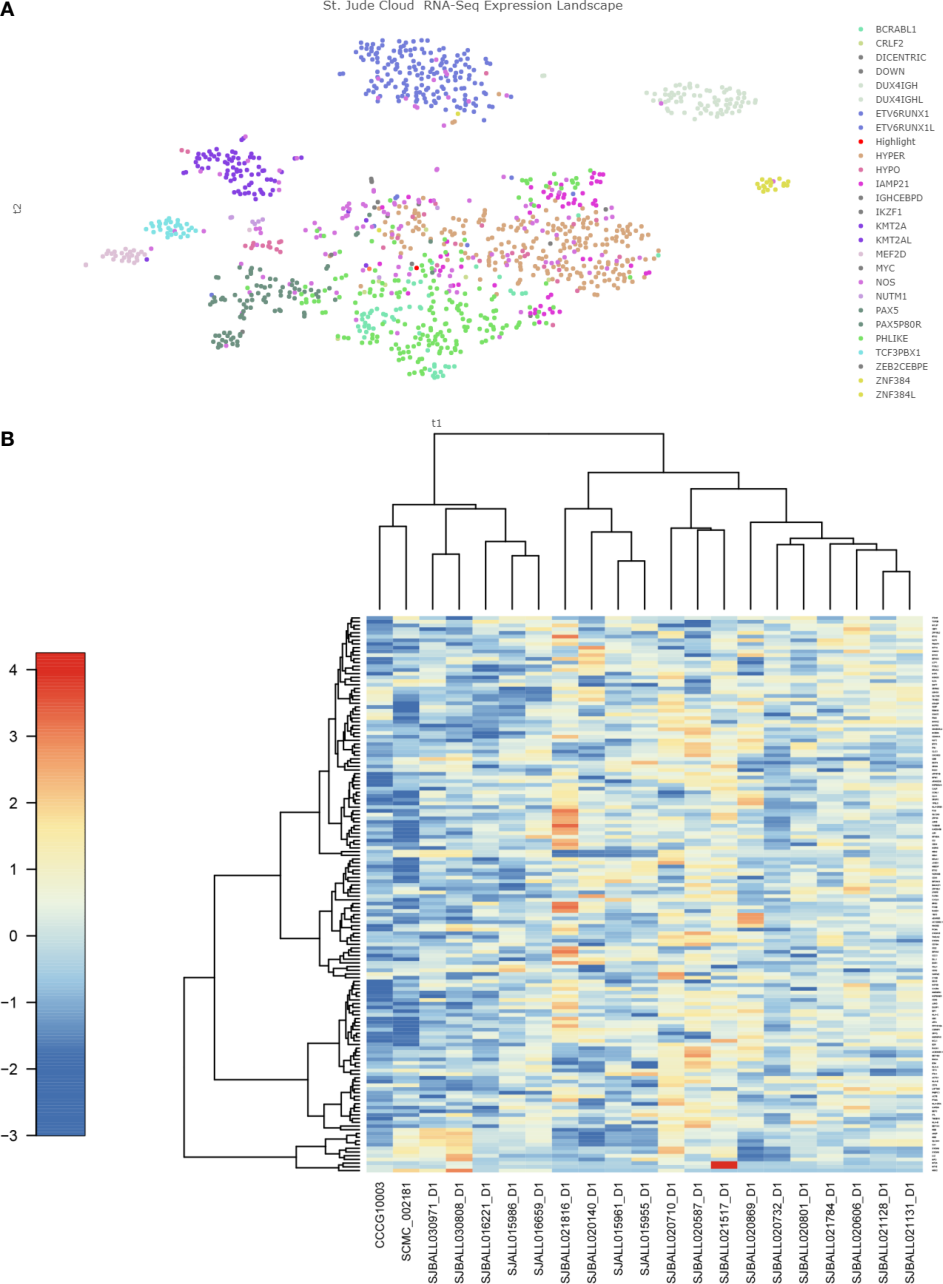
Gene expression characteristics of TCF4-ZNF384 **(A)** The red highlights are the two children with this TCF4-ZNF384, which can be seen not clustered with the other children with ZNF384-fusion. **(B)** Heatmap of the top 100 genes expressed in TCF4-ZNF384(N=2) and other ZNF384 subtypes (N= 19), as shown in the figure, these two patients are also relatively independent.

However, cluster analysis showed that the genetic characteristics of TCF4-ZNF384 were not clustered together with other ZNF384-rearrangement, and the characteristics of these two children were relatively independent in the Heatmap (**Figure 3**). In addition, during clinical treatment and follow-up, we found that the clinical treatment of these two children was very smooth. There were few adverse events and a complete remission was maintained in the evaluation and monitoring of bone marrow, which seemed to support the unique clinical history of this subtype of children and even predicted a good prognosis. This is what we learned about this new subtype through RNA.seq, and it’s because of technological innovations that we can more accurately assess the risk of treatment in children. Unfortunately, due to the small number of cases, further experimental verification and clinical follow-up are needed.

Studies have shown that, the immunophenotype of these children is often determined by the fusion partner of ZNF384 rather than by ZNF384 itself. For example, children with B-ALL carrying EP300-ZNF384 often have low or noexpression of CD10, accompanied by significant expression abnormalities of CD13 and/or CD33. Specific immunophenotypes have also been observed in other children with ZNF384 fusion, but the underlying genetic mechanisms need to be elucidated (3, 9).Previous studies have shown that ZNF384-FGS positive B-All has a unique transcriptomic gene ex-pression profile, which is associated with abnormal expression of myeloid antigens CD13 and/or CD33 (10, 11).Interestingly, there was no abnormal expression of CD13 or CD33 in case-1 and high expression of CD33 in case-2, and low expression of CD10 in both cases, and no other genes clearly associated with B lymphocyte differentiation were detected in transcriptome sequencing. At present, there are few systematic studies on ZNF384- rearrangement B-ALL, and there are limited literature reports on the prognosis of these patients. The possible reasons for this difference are still unknown.

In summary, this case illustrates the recognition of TCF4-ZNF384 in the diagnosis of ALL in children. The incidence of ZNF384 rearrangement of ALL in children is not high, and it is difficult to detect by conventional genetic and molecular biological tests (such as chromosome tests and FISH tests). However, RNA. seq can often detect these hidden fusions. Since the ZNF384 specific partner genes are closely related to the clinical outcome of the children, the identification of ALL patients with ZNF384 rearrangement by RNA.seq and other techniques is crucial to elucidate the results related to its specific fusion and develop appropriate therapeutic strategies. Finally, detection of this unique subtype and defining clinical characteristics may help better identify treatment options for patients.

## Data availability statement

The datasets presented in this study can be found in online repositories. The names of the repository/repositories and accession number(s) can be found below:

GSA human repository with accession HRA 002231.

## Ethics statement

All participants gave written informed consent prior the study, and the protocol was authorized by the Ethics Committee of the Second Affiliated Hospital of Anhui Medical University (No.201501). Written informed consent to participate in this study was provided by the participants’ legal guardian/next of kin.

## Author contributions

All authors listed have made a substantial, direct, and intellectual contribution to the work and approved it for publication.

## Acknowledgments

The authors would like to thank Dr. Liu Yu for their technical assistance, Shanghai Children’s Medical Center Translational Laboratory for their excellent data management and experimental assistance.

## Conflict of interest

The authors declare that the research was conducted in the absence of any commercial or financial relationships that could be construed as a potential conflict of interest.

## Publisher’s note

All claims expressed in this article are solely those of the authors and do not necessarily represent those of their affiliated organizations, or those of the publisher, the editors and the reviewers. Any product that may be evaluated in this article, or claim that may be made by its manufacturer, is not guaranteed or endorsed by the publisher.

## References

[1] Swerdlow SH Campo E Pileri SA Harris NL Stein H Siebert R. The 2016 revision of the world health organization classification of lymphoid neoplasms. Blood (2016) 127(20):2375–90. doi: 10.1182/blood-2016-01-643569 PMC4874220 26980727

[2] Langstein J Milsom MD Lipka DB. Impact of DNA methylation programming on normal and pre-leukemic hematopoiesis. Semin Cancer Biol (2018) 51:89–100. doi: 10.1016/j.semcancer.2017.09.008 28964938

[3] Liu YF Wang BY Zhang WN Huang JY Li BS Zhang M. Genomic profiling of adult and pediatric b-cell acute lymphoblastic leukemia. EBioMedicine (2016) 8:173–83. doi: 10.1016/j.ebiom.2016.04.038 PMC4919728 27428428

[4] Lilljebjörn H Henningsson R Hyrenius-Wittsten A Olsson L Orsmark-Pietras C Von Palffy S. Identification of ETV6-RUNX1-like and DUX4-rearranged subtypes in paediatric b-cell precursor acute lymphoblastic leukaemia. Nat Commun (2016) 7:11790. doi: 10.1038/ncomms11790 27265895 PMC4897744

[5] Shago M Abla O Hitzler J Weitzman S Abdelhaleem M. Frequency and outcome of pediatric acute lymphoblastic leukemia with ZNF384 gene rearrangements including a novel translocation resulting in an ARID1B/ZNF384 gene fusion. Pediatr Blood Cancer (2016) 63(11):1915–21. doi: 10.1002/pbc.26116 27392123

[6] Hirabayashi S Ohki K Nakabayashi K Ichikawa H Momozawa Y Okamura K. ZNF384-related fusion genes define a subgroup of childhood b-cell precursor acute lymphoblastic leukemia with a characteristic immunotype. Haematologica (2017) 102(1):118–29. doi: 10.3324/haematol.2016.151035 PMC5210242 27634205

[7] Zhou X Edmonson MN Wilkinson MR Patel A Wu G Liu Y. Exploring genomic alteration in pediatric cancer using protein-paint. Nat Genet (2016) 48(1):4–6. doi: 10.1038/ng.3466 26711108 PMC4892362

[8] Gocho Y Kiyokawa N Ichikawa H Nakabayashi K Osumi T Ishibashi T. A novel recurrent EP300-ZNF384 gene fusion in b-cell precursor acute lymphoblastic leukemia. Leukemia (2015) 29(12):2445–8. doi: 10.1038/leu.2015.111 25943178

[9] Yasuda T Tsuzuki S Kawazu M Hayakawa F Kojima S Ueno T. Recurrent DUX4 fusions in b cell acute lymphoblastic leukemia of adolescents and young adults. Nat Genet (2016) 48(5):569–74. doi: 10.1038/ng.3535 27019113

[10] Nyquist KB Thorsen J Zeller B. Identification of the TAF15-ZNF384 fusion gene in two new cases of acute lymphoblastic leukemia with a t (12;17) (p13; q12). Cancer Genet (2011) 204(3):147–52. doi: 10.1016/j.cancergen.2011.01.003 21504714

[11] Barber KE Harrison CJ Broadfield ZJ Stewart AR Wright SL Martineau M. Molecular cytogenetic characterization of TCF3 (E2A)/19p13.3 rearrangements in b-cell precursor acute lymphoblastic leukemia. Genes Chromosomes Cancer (2007) 46(5):478–86. doi: 10.1002/gcc.20431 17311319

